# Purpureocillium lilacinum Fungemia Masquerading as an Unidentified Yeast in a Heavily Immunosuppressed Patient With Relapsed Multiple Myeloma and Therapy-Related Mixed-Phenotype Acute Leukemia: A Case Report

**DOI:** 10.7759/cureus.116409

**Published:** 2026-09-17

**Authors:** Mostafa M Abouzahra, Enas Abdallah, Carolyn K Lackore, Namisha Kathuria, John Greene

**Affiliations:** 1 Faculty of Medicine, Al-Azhar University, Cairo, EGY; 2 Malignant Hematology, Moffitt Cancer Center, Tampa, USA; 3 Microbiology, Moffitt Cancer Center, Tampa, USA; 4 Internal Medicine, Government Medical College and Hospital, Chandigarh, IND; 5 Internal Medicine, Moffitt Cancer Center, Tampa, USA

**Keywords:** antifungal resistance, blood culture, fungemia, hematologic malignancy, immunocompromised host, neutropenia, paecilomyces lilacinus, purpureocillium lilacinum, voriconazole, yeast mimicry

## Abstract

*Purpureocillium lilacinum* (formerly *Paecilomyces lilacinus*) is an environmental saprophytic mold and an increasingly recognized opportunistic pathogen in immunocompromised hosts, most often causing cutaneous, ocular, or sinopulmonary disease; bloodstream infection (fungemia) is rare. The initial microscopic morphology on Gram stain of a positive blood culture can simulate yeast, delaying correct identification and appropriate antifungal therapy.

We describe a 67-year-old man with relapsed multiple myeloma and therapy-related mixed-phenotype acute leukemia, further complicated by a prior *Mycobacterium abscessus* vertebral epidural abscess, who was admitted with neutropenic fever, hypoxic *Burkholderia cenocepacia* pneumonia, and multiorgan dysfunction. Blood culture demonstrated a yeast-like organism that could not be identified as any common *Candida* species by rapid multiplex polymerase chain reaction (PCR). The patient was later found to have port-associated *Paecilomyces*/*Purpureocillium* fungemia, with only one day of documented fungemia. The port was removed, and repeat blood cultures were negative. The isolate was submitted to a reference mycology laboratory and was identified as *P. lilacinum* approximately two weeks after the index blood culture. Susceptibility testing reported resistance to amphotericin B and a high minimum inhibitory concentration (MIC) to itraconazole, with lower MICs to posaconazole and voriconazole. He received posaconazole and liposomal amphotericin B briefly, followed by voriconazole, with clinical improvement and no recurrent fungemia.

This case illustrates a clinically important diagnostic pitfall: rare filamentous molds such as *P. lilacinum* can mimic yeast morphologically and evade rapid yeast-identification panels, leading to delayed diagnosis in critically ill, heavily immunosuppressed patients. Early involvement of a reference mycology laboratory with molecular sequencing capabilities, when phenotypic identification is inconclusive, is warranted. The possibility of an amphotericin B-resistant mold simulating “yeast” fungemia should be considered in high-risk hematology-oncology patients.

## Introduction

*Purpureocillium lilacinum* is a filamentous, saprophytic mold that is ubiquitous in soil, decaying vegetation, and water, and is used commercially as a biocontrol agent against plant-parasitic nematodes. It was reclassified from the genus *Paecilomyces* in 2011 on the basis of molecular phylogenetic data, and it is present in much of the older clinical literature as *Paecilomyces lilacinus* [1]. Human infections are uncommon but increasingly reported, particularly among patients with hematologic malignancy, solid organ or hematopoietic stem cell transplantation, corticosteroid or other immunosuppressive therapy, and diabetes mellitus [2,3]. Reported manifestations range from keratitis, cutaneous and subcutaneous infection, and sinusitis to peritonitis, osteomyelitis, pulmonary disease, and, rarely, fungemia [2,4]. 

A clinically important diagnostic feature of *P. lilacinum* is that it can appear yeast-like early in growth, including in blood culture broth, before developing typical hyphae and conidiophores [1,5]. Because rapid blood-culture identification panels are designed mainly to identify common *Candida* species, *P. lilacinum* may initially be reported only as an unidentified “yeast.” This can delay recognition of the organism until the culture matures or molecular identification is performed. We report this phenomenon in a patient with a complex hematologic malignancy course and discuss the diagnostic and therapeutic implications.

The largest available synthesis of the condition, a combined analysis of 101 cases from the FungiScope® registry and the published literature, found that hematologic and oncologic disease was the leading predisposing factor (30.7% of cases) for infection, followed by corticosteroid treatment (26.7%), solid organ transplantation (25.7%), and diabetes mellitus (18.8%) [4]. Skin and lungs were the most frequently affected sites (36.6% and 25.7% of cases, respectively), with disseminated disease occurring in approximately one-fifth of patients. The overall mortality reported was 21.8%, which increased substantially among patients treated with amphotericin B monotherapy [4]. Notably, *P. lilacinum* accounted for breakthrough infection in about 10% of patients in that cohort, occurring despite ongoing antifungal therapy directed at other pathogens [4], a pattern broadly consistent with the wider literature on breakthrough invasive fungal infections in high-risk hematology patients, in which non-*Aspergillus*, non-Mucorales molds such as *P. lilacinum* remain comparatively uncommon but clinically consequential outliers [6]. 

Beyond individual case reports, *P. lilacinum* has recently drawn renewed public health attention through healthcare-associated keratitis and cutaneous pseudo-outbreak investigations linked to environmental or procedural sources [7,8]. Although our patient's fungemia was unrelated to these clusters, these reports underscore the organism’s evolving relevance to clinical microbiology and infection prevention. 

## Case presentation

Background and oncologic history 

A 67-year-old man had a history of multiple myeloma diagnosed approximately 15 years earlier, with remission achieved roughly 11 years before this presentation following autologous hematopoietic stem cell transplantation and subsequent maintenance therapy. Several months before the index presentation, he was found to have therapy-related mixed-phenotype acute leukemia, with blasts co-expressing myeloid (myeloperoxidase) and B-lymphoid (CD19) markers and harboring an FLT3 mutation. He was treated with induction therapy combining azacitidine, venetoclax, and gilteritinib with intrathecal chemotherapy. His course was complicated by new-onset atrial flutter and poor therapeutic tolerance. Therefore, therapy was subsequently simplified to gilteritinib and venetoclax because of poor tolerance. In the months preceding this admission, his course was complicated by relapse of both leukemia and multiple myeloma, with consideration being given to bispecific or CAR-T-directed therapy.

His history was also notable for prior *Mycobacterium abscessus* vertebral osteomyelitis with epidural abscess and cauda equina syndrome approximately one year earlier, requiring surgical debridement and prolonged multidrug antimycobacterial therapy. Additional comorbidities included paroxysmal atrial fibrillation/flutter, prior pulmonary embolism on apixaban, chronic kidney disease, and chronic lower-extremity edema. 

Presentation 

The patient was admitted with dyspnea, hypoxia, and clinical decompensation in the setting of leukopenia and profound immunosuppression from relapsed hematologic malignancy and ongoing therapy. Pertinent laboratory findings during hospitalization are summarized in Table 1, demonstrating leukopenia, anemia, and elevated lactate dehydrogenase.

**Table 1:** Pertinent Laboratory Findings During Hospitalization WBC: white blood cell count; AST: aspartate aminotransferase; ALT: alanine aminotransferase; LDH: lactate dehydrogenase

Laboratory Parameter	Patient Value	Reference Range	Interpretation
WBC (k/µL)	3.52	4.00-10.90	Low
Hemoglobin (g/dL)	8.8	11.4-15.0	Low
Platelet count (k/µL)	146	143-382	Within reference range
AST (U/L)	19	11-34	Within reference range
ALT (U/L)	<7	0-55	Within reference range
LDH (U/L)	323	135-225	High

Computed tomography of the chest demonstrated a right lower lobe consolidation with ground-glass opacity in the right middle and upper lobes, and he was treated empirically for pneumonia. Respiratory culture grew *Burkholderia cenocepacia*, which was treated with meropenem followed by trimethoprim-sulfamethoxazole; susceptibility testing was not performed. His course was complicated by encephalopathy and clinical deterioration in the setting of active malignancy, profound immunosuppression, and infection. He required intensive care unit admission with mechanical ventilation, vasopressor support, and continuous renal replacement therapy. 

Broad-spectrum antibacterial and antifungal therapies were initiated, including meropenem and micafungin, as well as continuation of azithromycin. Bronchoalveolar lavage culture grew *Candida tropicalis* and *Candida glabrata*, with a low-level positive galactomannan. Additional molecular testing detected *C. glabrata* and *C. tropicalis*, as well as *Actinomyces* and *Rothia* species, organisms more typically representing oropharyngeal microbiota than true pathogens in this context. 

Diagnostic course of the bloodstream isolate

Blood cultures were obtained because of persistent fever and clinical instability. The Gram stain of the positive blood culture bottle initially demonstrated yeast-like forms (Figure 1A). A rapid multiplex polymerase chain reaction (PCR)-based yeast identification panel was performed directly on the blood culture and excluded *Candida albicans, C. auris, C. glabrata, Candida krusei, Candida parapsilosis, C. tropicalis,* and *Cryptococcus neoformans*/*Cryptococcus *gattii. The provisional blood culture report characterized the organism as an unidentified ("yeast - not any of the above by PCR"). Only one day of fungemia was documented. The port was removed for source control, and repeat blood cultures were negative. Upon prolonged incubation on solid media, the organism developed filamentous growth with lilac colony morphology (Figure 1B). The provisional identification was updated to probable *P. lilacinum* based on colonial morphology and microscopic identification of rudimentary penicillus structures with tapering phialides and round conidia, along with hyaline septate hyphae (Figure 1C). The isolate was sent to a reference mycology laboratory for identification confirmation and antifungal susceptibility testing. Approximately two weeks after the index blood culture, the reference laboratory confirmed the identification as *P. lilacinum*.

**Figure 1:**
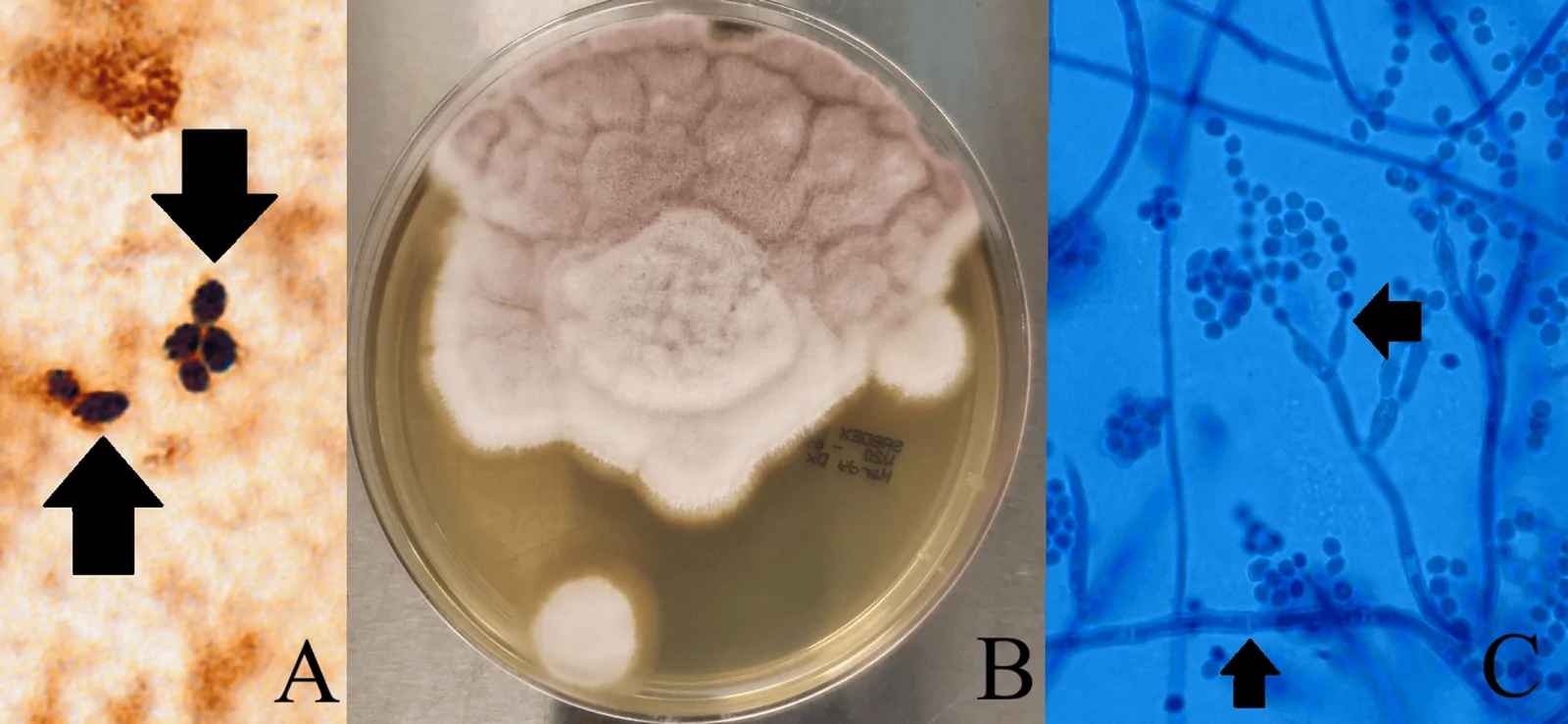
Microbiologic Findings of the Bloodstream Isolate (A) Gram stain from the positive blood culture bottle demonstrating yeast-like forms. (B) Colony morphology of *Purpureocillium lilacinum* showing a distinctive lilac color. (C) Microscopic morphology of *P. lilacinum* demonstrating rudimentary penicillus structures with tapering phialides (side arrow) producing round conidia, along with hyaline septate hyphae forming the mycelium (up arrow).

Antifungal susceptibility testing reported resistance to amphotericin B (minimum inhibitory concentration (MIC) ≥ 8 µg/mL) and a high MIC to itraconazole (MIC ≥ 8 µg/mL), with substantially lower MICs to posaconazole (0.5 µg/mL) and voriconazole (1 µg/mL), a pattern consistent with previously reported susceptibility profiles for this organism [2,9]. 

The laboratory timeline is summarized in Table 2.

**Table 2:** Timeline From Index Blood Culture to Definitive Identification of the Bloodstream Isolate PCR: polymerase chain reaction; MIC: minimum inhibitory concentration; MEC: minimum effective concentration

Hospital Day	Laboratory Event	Result/Finding
Day 0	Index blood culture obtained for fever	Bottle incubated; no growth reported for the first 3 days
Day 3	Positive blood culture bottle flagged; Gram stain performed	Yeast forms identified on Gram stain
Day 3	Rapid multiplex blood-culture PCR panel	"Yeast - not *Candida albicans, Candida auris, Candida glabrata, Candida krusei, Candida parapsilosis, Candida tropicalis, *or* Cryptococcus neoformans*/*Cryptococcus* *gattii*"
Day 8	Repeat testing of the same blood-culture isolate	Same non-*Candida* “yeast” result reported
Day 13	Subculture matured on solid media	Filamentous growth observed; reported as "probable *Purpureocillium lilacinum*"; isolate sent to reference mycology laboratory
Day 14	Reference laboratory receipt	Identification and susceptibility testing pending
Day 15	Reference laboratory, preliminary report	*Purpureocillium lilacinum*; susceptibility testing to follow
Day 17	Reference laboratory, final report	*Purpureocillium lilacinum* confirmed. Susceptibility: amphotericin B MIC ≥ 8 µg/mL, reported resistant; anidulafungin MEC ≥ 8 µg/mL; caspofungin MEC ≥ 8 µg/mL; micafungin MEC 2 µg/mL; itraconazole MIC ≥ 8 µg/mL; posaconazole MIC 0.5 µg/mL; voriconazole MIC 1 µg/mL.

Treatment and outcome

Initial antifungal therapy included micafungin. After the yeast-like blood-culture result was reported, the patient was contacted to return for further antifungal management. He continued posaconazole before readmission, received liposomal amphotericin B briefly during the subsequent hospitalization, underwent port removal for source control, and was then transitioned to voriconazole. Although posaconazole had the lowest MIC for this isolate, voriconazole was used because of its broad-spectrum mold activity, oral availability for outpatient therapy, and greater likelihood of insurance coverage. Venetoclax was stopped on admission, and azole therapy was coordinated with the patient’s ongoing gilteritinib therapy because of CYP3A4 drug-drug interaction considerations. QTc monitoring was performed during concomitant azole and gilteritinib therapy.

Chest CT demonstrated atelectasis and pleural effusions without pulmonary nodules to suggest a primary pulmonary source of the mold infection or septic emboli (Figures 2A-2B).

**Figure 2:**
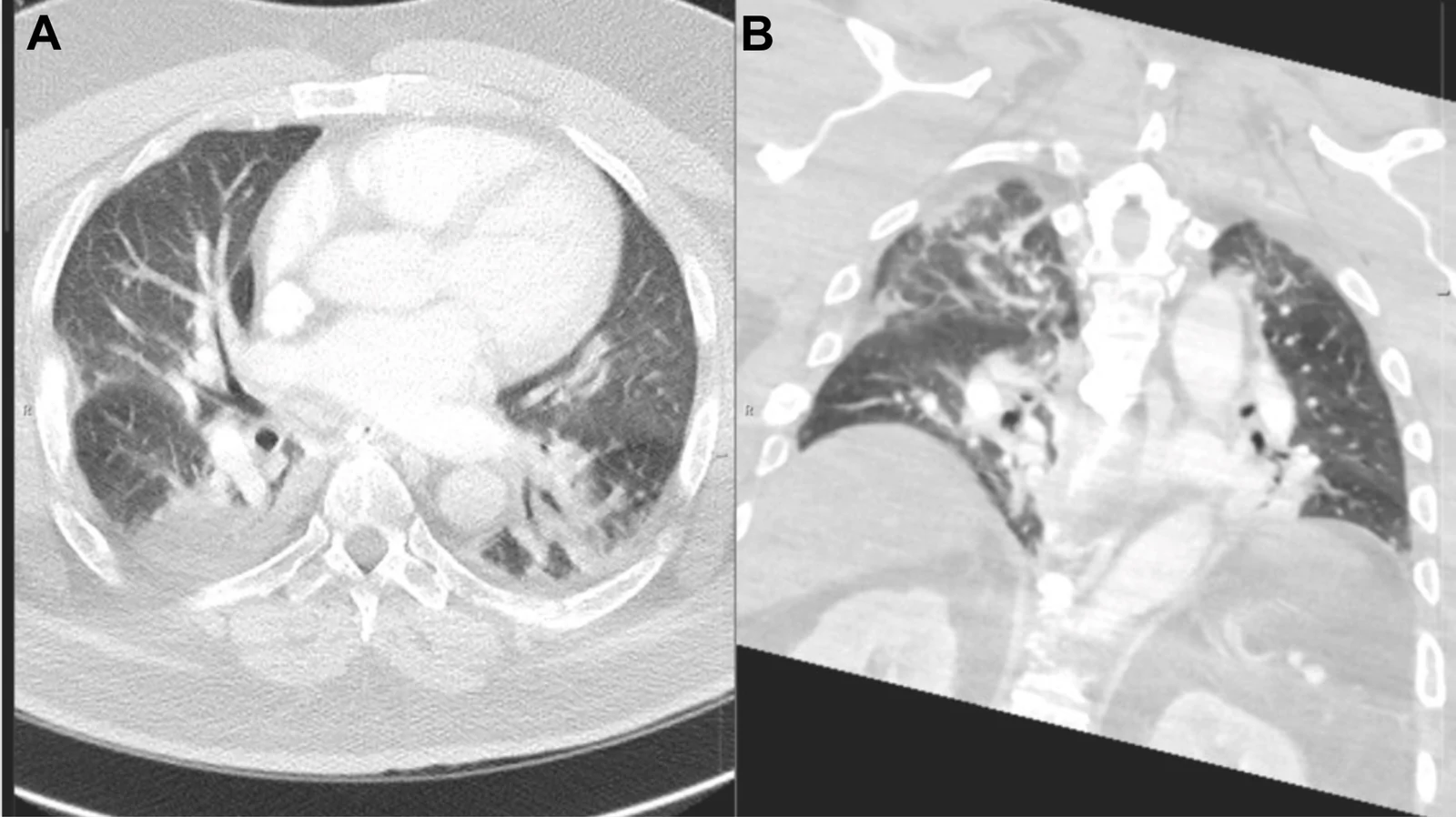
Chest Computed Tomography Findings (A) Axial view and (B) coronal view demonstrating atelectasis and pleural effusions without pulmonary nodules, cavitary lesions, or findings suggestive of pulmonary mold infection or septic emboli.

There was no clinical or radiographic evidence of deep-organ dissemination, including involvement of the liver, spleen, or brain. A small right-leg nodular lesion had been documented, but it was not clinically attributed to *Purpureocillium* infection. Thus, the presentation was most consistent with isolated, port-associated fungemia without evidence of confirmed deep-organ dissemination. 

Given clinical improvement, port removal, and negative repeat blood cultures, antifungal therapy was continued with voriconazole under infectious disease and hematology-oncology co-management. The patient subsequently improved clinically, was discharged home, and had no evidence of recurrent fungemia at outpatient follow-up. 

## Discussion

*P. lilacinum* is an emerging opportunistic mold pathogen whose clinical significance has been increasingly recognized in immunocompromised populations, particularly patients with hematologic malignancy, solid organ or stem cell transplantation, and chronic immunosuppressive therapy [2,3]. In the largest published cohort to date - 101 cases compiled through the FungiScope® registry and a literature review - hematologic malignancy and neutropenia were among the most consistently identified host risk factors for invasive disease, and fungemia, while uncommon relative to cutaneous or ocular disease, carried substantial morbidity [4]. Our patient exemplifies this risk profile: relapsed multiple myeloma, therapy-related mixed-phenotype leukemia under active cytotoxic and targeted therapy, profound and prolonged immunosuppression, and a preceding history of an unrelated but similarly opportunistic infection (*M. abscessus* vertebral osteomyelitis), reflecting a cumulative, multi-year burden of immune compromise.

The central diagnostic lesson from this case is the propensity of *P. lilacinum* to mimic a yeast on initial blood culture evaluation. Under certain growth conditions, including within blood culture broth, this mold can produce swollen, budding, or chain-like cellular forms that are morphologically indistinguishable from yeast on Gram stain before hyphal elements become apparent on subculture [1,5]. Because contemporary rapid blood-culture identification platforms are largely designed to differentiate common *Candida* species (and, on some panels, *Cryptococcus*), an isolate of *P. lilacinum* would typically be reported only as an unidentified, non-*Candida* "yeast," as occurred here. This can create a diagnostic and therapeutic gap of days to weeks while the organism is subcultured and grown to maturity and identified and/or referred for phenotypic and/or molecular (e.g., ITS or β-tubulin gene sequencing) identification at a reference laboratory [1]. Our team's prior institutional experience with a similarly delayed identification of a *Scedosporium* species from blood culture underscores that this is not an isolated phenomenon but a recurring pitfall with several genera of hyaline molds capable of yeast-like growth in blood culture systems. 

The treatment implications of this diagnostic delay are significant. *P. lilacinum* is intrinsically resistant to amphotericin B and frequently resistant to itraconazole, while newer triazoles, such as voriconazole and posaconazole, generally retain lower MICs and are regarded as the most active available agents. This was consistent with the antifungal susceptibility pattern observed in our patient [2,4,9]. Multiple case reports describe clinical cure of *P. lilacinum* fungemia or disseminated disease with voriconazole, including in patients who had failed or were intolerant of amphotericin B-based regimens [9,10]. However, no antifungal regimen has been validated in controlled trials; source control such as catheter or port removal is emphasized whenever feasible, and reported mortality in disseminated disease among heavily immunocompromised hosts remains substantial [2,4]. In this case, clearance likely reflected the combination of source control with port removal, negative repeat blood cultures, and subsequent azole therapy guided by the susceptibility profile.

Because species-specific clinical breakpoints are not established for *P. lilacinum*, MIC and minimum effective concentration (MEC) values were interpreted cautiously and in the context of published susceptibility patterns, source control, host factors, and clinical response. MIC values were reported for amphotericin B and triazoles, whereas MEC values were reported for echinocandins. No CLSI or EUCAST species-specific clinical breakpoints were available for most agents in this case.

Although bronchoalveolar lavage culture and additional molecular testing detected *C. glabrata* and *C. tropicalis*, these findings did not identify the bloodstream mold isolate and were of uncertain clinical significance in this context. The *Candida* species detected in bronchoalveolar lavage and molecular respiratory testing were not recovered from blood cultures and did not correspond to the bloodstream isolate, which later matured as a filamentous mold and was confirmed as *P. lilacinum*. Therefore, these respiratory findings were interpreted as being of uncertain clinical significance in relation to the fungemia. Definitive diagnosis of the fungemia required culture maturation, reference mycology laboratory identification, and susceptibility testing.

Although our patient was not receiving mold-active antifungal prophylaxis at the time blood cultures were drawn, this case fits the broader clinical context of rare mold infections in heavily treated hematology patients. Breakthrough invasive fungal infection reviews show that uncommon molds such as *Purpureocillium*, *Scedosporium*, and *Lomentospora* remain a small but important minority of cases in high-risk hematology populations [6]. Therefore, persistent or atypical infectious syndromes in high-risk immunocompromised patients should prompt consideration of rare molds when routine diagnostics are inconclusive.

Advances in fungal identification technology may help narrow the diagnostic window illustrated in Table 2. Matrix-assisted laser desorption/ionization time-of-flight mass spectrometry (MALDI-TOF MS) has transformed bacterial and yeast identification directly from blood culture broth, reducing turnaround time from days to minutes once adequate biomass is available, but its application to filamentous molds, including *Purpureocillium*, has lagged behind that for yeasts because of sparser reference spectral libraries and the requirement for mature, sporulating growth before reliable spectra can be obtained [11]. In practice, this means that even laboratories equipped with MALDI-TOF MS may not shorten the identification timeline for an organism like *P. lilacinum* unless paired with molecular sequencing (for example, of the internal transcribed spacer region or β-tubulin gene) performed early, rather than only after conventional phenotypic methods have been exhausted. Earlier engagement of a reference mycology laboratory once an isolate fails to be identified as a common yeast species, rather than waiting for full phenotypic maturation on solid media, may meaningfully shorten time to targeted antifungal therapy in similarly high-risk patients.

This diagnostic pitfall is not unique to *P. lilacinum*. Similar yeast-like blood-culture mimicry has been described with *Lomentospora prolificans *and *Scedosporium prolificans* in immunocompromised hosts [12,13]. These reports, together with our case, support maintaining rare molds in the differential diagnosis when a high-risk immunocompromised patient has an unidentified, non-*Candida* yeast-like bloodstream isolate and routine identification stalls. 

Limitations 

This report is limited by its retrospective, single-patient design and by residual uncertainty regarding the clinical significance of concurrent pulmonary *Candida* species. Although the episode was clinically managed as port-associated fungemia with port removal and negative repeat blood cultures, definitive exclusion of other occult portals of entry is limited by the retrospective nature of the report.

## Conclusions

*P. lilacinum* should be considered when a yeast-like blood-culture isolate from a high-risk immunocompromised patient cannot be identified as a common *Candida* species by rapid molecular testing. This case highlights the importance of early reference mycology laboratory involvement, source control for port-associated fungemia, and susceptibility-guided antifungal therapy given the organism’s intrinsic resistance to amphotericin B and the need for triazole selection based on susceptibility testing.
